# Bifidobacteria Encapsulation and Viability of Probiotic Culture during Oral Delivery in a Milk Drink Matrix

**DOI:** 10.1155/2023/8484835

**Published:** 2023-07-27

**Authors:** Tatiana Voblikova, Kristina Laricheva

**Affiliations:** Yaroslav-the-Wise Novgorod State University, 173003 Veliky Novgorod, Russia

## Abstract

The use of an alginate hydrogel exclusively for the immobilization of bifidobacteria during oral delivery led to a decrease in the total number of bifidobacteria to 4.0 lg CFU/ml in pH gradients in models of the stomach and intestines, which required clarification of the composition of the protective coating. The introduction of resistant starch into the composition of the microcapsule contributed to the preservation of the viability of immobilized bifidobacteria up to 87% of the initial concentration when passing through the model environment simulating the human digestion system. The introduction of sodium carboxymethylcellulose into the composition of the hydrogel contributed to the regulation of the degradation of the polymer matrix and the controlled release of bifidobacteria. The use of sodium carboxymethylcellulose 0.5% in the formation of a polymer microcapsule provided the maximum encapsulation efficiency of 93.2% and the maximum decay rate of bacteria-loaded microcapsules of 95.7%. The modified alginate matrix contributes to maintaining the level of viable cells of probiotic microorganisms (*Bifidobacterium bifidum* 791) of at least 10^8^ CFU/g when stored for three weeks. As a result of the research, a system for oral delivery of immobilized bifidobacteria in the structure of microparticles with a closed surface in the matrix of a milk drink has been developed, which increases the effectiveness of probiotics for human health in the composition of food products.

## 1. Introduction

The intestinal microbiota plays an essential role in metabolic, nutritional, physiological, and immunological processes in the human body. In recent years, there has been a dramatic increase in the number of studies focusing on the gut microbiota, which confirm the close relationship between the gut microbiota and human health. The gastrointestinal tract performs not only a digestive but also an immune function; in particular, it is involved in the implementation of the body's defense reactions against pathogenic, opportunistic microorganisms and many inorganic substances. Since the gut microbiota is known to play an important role in the human body, manipulations with these microorganisms using probiotics, prebiotics, and synbiotics are a promising approach to improve and maintain health [1–3].

Starting from the oral cavity, probiotic cultures in food products begin their way through the gastrointestinal tract, where they are exposed to various physical and chemical influences, due to which they can completely lose their biological activity.

Some of the negative impacts of oral delivery of active ingredients can be overcome by micro- and nanoencapsulation techniques that could bring new delivery systems to the food industry to fortify foods and beverages with probiotics. Micro- and nanoencapsulation of nutraceuticals can provide a number of advantages in terms of the stability of administered active ingredients.

Probiotic microorganisms are included in a wide range of foods, dietary supplements, and pharmaceuticals to improve human health and well-being. Probiotics must be present at ≥10^6^ CFU/g in a food product prior to the time of consumption to provide a beneficial effect on human health [4–7]. However, maintaining the viability of bacterial cells during storage and passage through the gastrointestinal tract remains an issue. Encapsulation of bifidobacteria in food-grade hydrogel particles potentially reduces their sensitivity to environmental stresses [8]. Hydrogels are macromolecular networks capable of absorbing and releasing aqueous solutions in a reversible manner in response to specific environmental influences.

To improve human health, probiotics must survive transit in the harsh environment of the stomach and be able to reach the intestine in sufficient quantities to ensure colonization and proliferation [9–11]. The health effectiveness of probiotics can be increased by the combined use of probiotics with a prebiotic growth agent that promotes the colonization of probiotics in the human digestive tract [12, 13].

The susceptibility of probiotics to low pH and high temperature limits their use. One suitable way to prevent the degradation of bioactive components is to encapsulate them. The development of effective microcapsules requires careful selection of methods and materials for microencapsulation [14–16].

Microencapsulation is a process in which probiotic cells are incorporated into an encapsulating matrix or membrane that can protect the cells from degradation by negative environmental factors and release at a controlled rate under certain conditions. The purpose of microencapsulation of probiotics is to protect them from low pH, bile salts, and other food constituents that they encounter during their passage through the gastrointestinal tract.

Encapsulating materials are generally considered safe ingredients that can be used in foods. To obtain microcapsules by various methods, mainly food polymers are used, such as alginate, chitosan, carboxymethylcellulose, xanthan gum, starch, carrageenans, gelatin, and pectin. In addition, there is a trend towards the use of encapsulation in milk proteins such as casein and whey protein [8, 17–23]. Alginate is a natural polymer that has been successfully applied as a pH-sensitive microencapsulation material due to its simplicity, nontoxicity, biocompatibility, and low cost. Alginate is a monovalent form of alginic acid belonging to a polysaccharide extracted from algae, composed of *β*-D-mannuronic and -L-guluronic acids. Different amounts and sequential distribution of *β*-D-mannuronic and -L-guluronic acids in the chain can affect the functional properties of alginate as an auxiliary material. Alginates undergo ionotropic gelation in an aqueous solution in the presence of divalent cations such as Ca2+ and Ba2+.

Alginates are mucoadhesive, but cross-linked alginates are usually brittle. The high porosity of alginate microcapsules limits their protective effect during heat treatment, gastric modeling, or the use of controlled release formulations [9]. Thus, the inclusion of other polymers is usually necessary to improve the thermal performance and stability of alginate-based microcapsules for use in food products. It has been found that the addition of xanthan gum to a chitosan-coated alginate system can minimize the loss of lactic acid bacteria *Lactobacillus plantarum* (<6%) at 90°C for 5 minutes.

Consequently, blending with mucoadhesive polymers is one of the most popular approaches for creating various cross-linked alginate mucoadhesive microcapsules for the controlled delivery of biologically active components. Blending with suitable polymers can improve the encapsulation of the biologically active component and its stability.

Cellulose is a long-chain natural polymer widely used as an excipient in the pharmaceutical industry. Because of its poor solubility, cellulose has been chemically modified into cellulose derivatives for broader applications. It has been found that incorporation of less hydrophilic cellulose derivatives, such as methylcellulose and hydroxypropyl methylcellulose, in alginate-based microspheres enhances drug encapsulation and drug release.

Sodium carboxymethylcellulose is one of the best known polymers from the cellulose family. Sodium carboxymethylcellulose is a water-soluble derivative of cellulose ether, consisting of *β*-linked glucopyranose residues with various levels of carboxymethyl substitution [24].

It is known that aqueous solutions of sodium carboxymethylcellulose improve the strength of the gel when heated. This correlates with the formation of an infinite network structure during polymer dehydration. This gel-forming property of sodium carboxymethylcellulose contributed to its use in various technological fields, in particular, in food, medical, and pharmaceutical industries [24].

Among the aforementioned cellulose ethers, only sodium carboxymethylcellulose is a polyelectrolyte and is sensitive to changes in pH and ionic strength. Indeed, the presence of sodium carboxymethylcellulose in a cellulose-based hydrogel provides the hydrogel itself with electrostatic charges attached to the network, which have a dual effect on swelling ability. On the one hand, the electrostatic repulsion that occurs between charges of the same sign causes the polymer chains to move into a more elongated state than in a neutral network, which increases swelling. On the other hand, the counterions that are present in the gel to provide macroscopic electrical neutrality induce more water to enter the network because of the Donnan-type effect. The osmotic pressure depends on different concentrations of mobile counterions between the gel and the external solution, which makes the gel sensitive to changes in pH or ionic strength. The polyelectrolyte nature of sodium carboxymethylcellulose makes it ideal for developing superadsorbent hydrogels with smart behavior [24].

The development of the optimal composition of the polymer shell for the immobilization of bifidobacteria and the preservation of their viability during oral delivery is an urgent issue, the solution of which will increase the effectiveness of the use of probiotics for human health in the matrix of dairy products.

The aim of the work is to establish in the course of research the optimal composition and structure of a microcapsule with a controlled release of bifidobacteria and the maximum efficiency of encapsulation, consisting of biodegradable polymers to maintain the viability of probiotic culture during storage and oral delivery in the matrix of a fermented milk drink.

## 2. Research Materials and Research Methods

### 2.1. Microorganism and Growth Conditions

*Bifidobacterium bifidum* 791 (preparation “Bifidobacterin” produced by CJSC “Ecopolis,” Kirov) and *Bifidobacterium bifidum* 791, immobilized in a polymer microcapsule, were used as objects of research.

To obtain a bacterial concentrate of bifidobacteria not lower than 10^9^ CFU/ml in liquid form, a nutrient medium was used for cultivating bifidobacteria. A sublimated culture of the *Bifidobacterium bifidum* 791 strain was added to the prepared nutrient medium and cultivated at a temperature of 37–38°C until a microbial mass was formed with a bifidobacteria content of at least 10^8^ CFU/ml. Next, the second passage was conducted. The second passage included the introduction of 10% of the grown culture of the *Bifidobacterium bifidum* 791 strain into the cultivation and accumulation medium at a temperature of 37-38°C. The third passage included the introduction of 10% of the grown culture of the *Bifidobacterium bifidum* 791 strain into the cultivation and accumulation medium at a temperature of 37-38°C for 6 hours. Next, centrifugation was performed at 4°C for 20 minutes at 5,000 rpm. The resulting bacterial concentrate contained at least 10^10^ CFU/ml. The resulting bacterial concentrate was used to obtain microcapsules.

### 2.2. Obtaining Microencapsulated Cells

Microcapsules were obtained in accordance with the technology of extrusion. The basis for obtaining microcapsules was a solution containing 1% sodium alginate+1% resistant starch+0.5% sodium carboxymethylcellulose. After complete dispersion of the polymers, the *Bifidobacterium bifidum* 791 strain was added to the solution and the multicomponent composition was sprayed into 0.1 M CaCl_2_ using an airbrush (model EW 110) with a nozzle size of 0.3 mm, connected to an air compressor (model Jas–1203). The resulting particles were stirred for 30 min in CaCl_2_ solution to ensure complete gelation and then removed from the solution.

### 2.3. Simulation of the Human Digestion Process and Assessment of the Survival of Bifidobacteria In Vitro

The method for studying the survival of probiotic microorganisms under in vitro conditions simulating the human digestion process consists in incubating microorganisms at a temperature of 37 ± 1°С successively in an acidic model medium with acidin-pepsin (pH 2.0) and an alkaline model medium with panzinorm forte 20000 (pH 6.8-5.8) during the average residence time of the mixed food in the stomach and intestines, respectively, with the subsequent determination of the number of surviving microorganisms by the formation of colony-forming units of bifidobacteria (CFU/ml) in the series of tenfold limiting dilutions, according to MUK 4.2.999-00, MUK 4.2.2602-10, GOST R 4.1.1672-03, and OFS “Determination of specific activity of probiotics.”

### 2.4. Evaluation of the Efficiency of Encapsulation

The granules (100 mg) were immersed in 10 ml pH 6.8 model medium with stirring at 200 rpm for 120 minutes for complete dissolution. The number of bifidobacteria was expressed in CFU per milliliter, and the encapsulation efficiency was calculated according to the following formula:

(1) $$\begin{array}{c} \text{Encapsulation efficiency}=\frac{\text{Lo}{\text{g}}_{10}\;N_{1}}{\text{Lo}{\text{g}}_{10}\;N_{2}}\times 100\%, \end{array}$$

where *N*_1_ is the initial amount of cell suspension added to the biopolymer mixture and *N*_2_ is the number of captured bacterial cells loaded inside the granules [25, 26].

The number of viable microorganisms was determined by the formation of colony-forming units of bifidobacteria (CFU/ml) in the rows of tenfold limiting dilutions.

**Figure 1:**
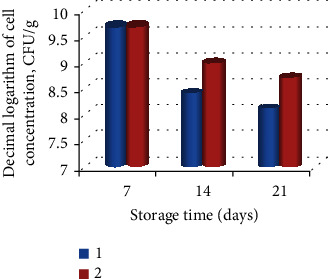
Survival of free and encapsulated *Bifidobacterium bifidum* 791 culture in a fermented milk drink during storage: (1) free and (2) encapsulated.

The study of the preservation of the viability of bifidobacteria during storage of a fermented milk drink from sheep's milk was carried out at a temperature of 4°C for 21 days (Figure 1). The fermented milk drink was produced by the tank method from sheep's milk pasteurized at 63°C for 30 minutes before preparation using *Streptococcus* ssp. *thermophilus* and *Lactobacillus delbrueckii* ssp. *bulgaricus*.

### 2.5. Morphological Assessment and Determination of the Average Diameter of Microcapsules

The overall morphology of the microcapsules was determined using a scanning electron microscope (SEM) (MIRA3, TESCAN). The microcapsules were placed on the substrate of the microscope stage using a double-sided tape coated with gold sputtering. The accelerating voltage of the microscope is 5 kV. Microcapsule diameters were determined using ImageJ software (NIH, USA). The average diameter was determined by measuring 100 microcapsules.

### 2.6. Statistical Analysis

All experiments were performed in at least three replications. The objectivity of the choice of the number of replications of experiments and the minimum sample size in obtaining the results of the presented studies was determined on the basis of statistical processing of experimental data providing a statistical reliability of 0.95 (or 95%) and a significance level of < 0.05 (or 5%). Statistical data processing was performed using the multifunctional software Statistica 6.0.

## 3. Results and Discussion

Oral intake of probiotics presents a number of problems, especially their vulnerability during gastric transit, low pH, and enzymatic attack [2].

To keep probiotics viable for effective colonization, the bacteria must be protected not only from acidic and enzymatic degradation by gastric fluid but also from the harmful effects of bile in the intestine during transit in the gastrointestinal tract. The microcapsules were tested for their stability in model environments simulating the human digestion system.

Encapsulated *Bifidobacterium bifidum* 791 was exposed to model environments simulating human digestion. To study the protective properties of microcapsules made of biodegradable polymers on bifidobacteria, solutions with pH gradient were used: pH 1.2, simulation of the stomach environment with the exposure for 30 to 120 min; pH 4.5, simulation of the duodenum environment with the exposure for 15 to 60 min; pH 6.8, simulation of the jejunum environment with the exposure for 60 to 120 min; pH 7.2, simulation of the ileum environment with the exposure for 60 to 120 min; and pH 5.8, simulation of the environment of the large intestine for up to 18 hours, at 37 ± 1°C with periodic stirring by circular movements of vials with test material. At each time point, sampling was performed to determine the survival of bifidobacteria in models of the stomach and intestines with titration by the method of tenfold serial dilutions from 10-9 to 10-1 CFU/ml in two parallel rows of test tubes. Bacterial inoculation was thermostatted at 37 ± 1°C for 72 h.

In the absence of significant differences in the number of CFU/ml of control and experimental samples, it was concluded that there was no effect of the test concentration of the substance of the model environment of the stomach and intestines in the in vitro model; in the case of a significant decrease in CFU/ml in the test samples, compared with the control, by at least one logarithmic order, a conclusion was made about the inhibitory effect of the substances in the in vitro model.

**Figure 2:**
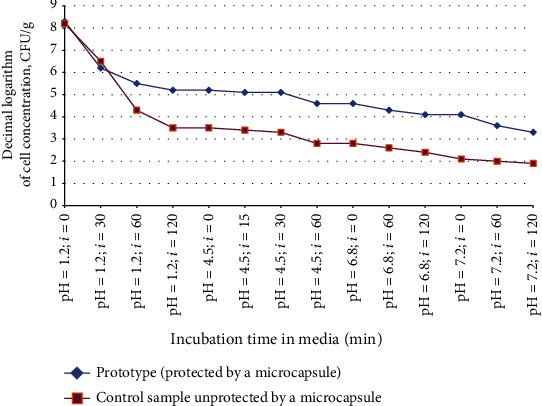
Survival of bifidobacteria under in vitro conditions simulating the process of human digestion.

The results of studying the survival of probiotic microorganisms under in vitro conditions simulating the process of human digestion are shown in Figure 2.

The obtained data shown in Figure 2 demonstrate a significant decrease in the number of viable bifidobacteria to 62.2% in the control sample not protected by an alginate-based microcapsule, while in the experimental sample of immobilized *Bifidobacterium bifidum* 791, in the structure of alginate hydrogel, the concentration of viable bifidobacteria is reduced only by 37.5% of the initial concentration when passing the model stomach environment (рН 1.2). This confirms the protective effect of the biodegradable natural polymer on bifidobacteria when passing them through an in vitro model of the stomach. However, reducing the total number of bacteria to 4.0 lg CFU/ml in acidity gradients in the stomach and intestine models required clarification of the composition of the protective coating.

Immobilization of bifidobacteria in alginate protects them from aggressive external factors. Microspheres obtained on the basis of alginate do not provide a sufficient degree of protection, as evidenced by the decrease in the viability of encapsulated bifidobacteria as a result of the passage through model environments of the stomach and intestines. These shortcomings can be effectively eliminated by mixing alginate with other polymers or by using a structural modification of alginate using various additives [27].

Alginate microcapsules were modified by introducing resistant starch into the protective matrix. The introduction of resistant starch into the structure of microcapsules improved the protection and preservation of the viability of microencapsulated bifidobacteria up to 87% of the initial concentration when passing through model environments simulating human digestion.

The data obtained are consistent with the results of some researchers who also reported a higher survival rate of bacteria in alginate microcapsules containing natural polymers (galactooligosaccharides/inulin, fructooligosaccharides, and monosaccharides) under simulated severe gastrointestinal conditions compared with alginate microparticles without prebiotics [8, 28].

**Figure 3:**
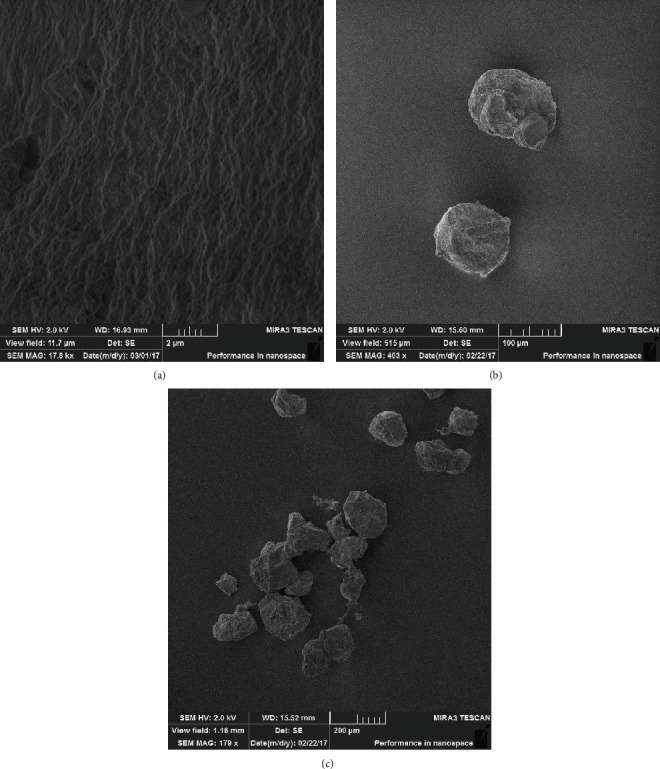
Morphology and microstructure of lyophilised microparticles (alginate+resistant starch), obtained using scanning electron microscopy: (a) microparticle surface; (b) microparticles (alginate+resistant starch); (c) general appearance of the particles (alginate+resistant starch).

The developed method for obtaining microcapsules based on biodegradable nontoxic polymers of natural origin made it possible to obtain microcapsules with a closed surface. The lyophilised microparticles had an average diameter of 150 *μ*m (alginate and resistant starch matrix) and 97 *μ*m (alginate matrix). The microstructure of the microcapsule surface is shown in Figure 3.

Some studies by other authors have characterized the alginate matrix as an oral delivery system with serious limitations, such as the rapid release of the immobilized active ingredient, caused by physical instability, and the high solubility of Ca-alginate microcapsules in neutral and weak alkaline media [29]. It is possible to overcome these limitations by applying different approaches to obtaining modified microcapsules by mixing and/or coating and by complexing polyelectrolyte with polymers [30–34].

The effect of sodium carboxymethylcellulose concentration in microcapsules on the release of encapsulated *Bifidobacterium bifidum* 791 was evaluated in developing a method of oral delivery of bifidobacteria.

**Figure 4:**
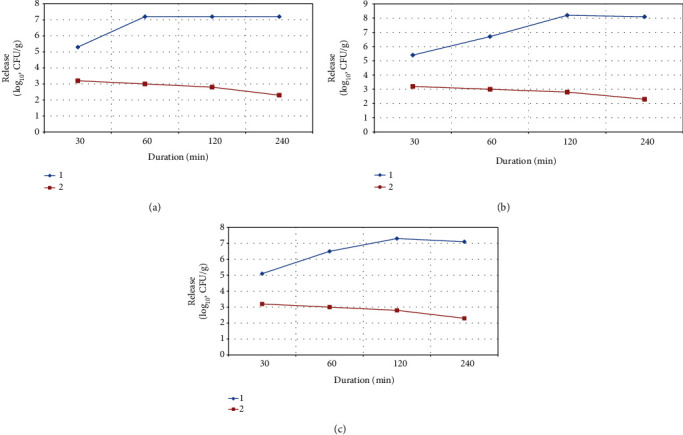
Dependence of the release of bifidobacteria at pH 6.8 on the content of Na-CMC 0.3% (a), 0.5% (b), and 1.0% (c) in the microcapsule structure: (1) immobilized bifidobacteria and (2) bifidobacteria unprotected by microcapsule.

Sodium carboxymethylcellulose was introduced into the microcapsule to regulate the degree of degradation of the polymeric matrix and the controlled release of bifidobacteria (Figure 4). The choice of the concentration range of the component composition of the microcapsule is determined by the maximum capacity and morphological characteristics of the microparticle, as well as by the efficiency of protection of bifidobacteria from external negative factors. Mixing polymers of alginate and sodium carboxymethylcellulose leads to the formation of strong inter and intramolecular hydrogen bonds between hydroxyl groups of polymer chains in polymeric microcapsules, which provides an increased degree of protection of bifidobacteria from external aggressive factors.

When sodium carboxymethylcellulose was added to the microcapsule matrix, an increase in the number of released viable microorganisms was observed after the first 30 min in phosphate buffer solution (pH 6.8), and the maximum decay of loaded microcapsules was observed after 120 min. A sodium carboxymethylcellulose concentration of 0.5% resulted in a maximum encapsulation efficiency of 93.2% and a maximum decomposition rate of 95.7% of the loaded microcapsules.

The study of the preservation of viability of bifidobacteria during storage of fermented milk drink from sheep's milk was conducted at 4°C for 21 days (Figure 1). The fermented milk drink was obtained by the tank method from sheep's milk pasteurized at 63°C for 30 minutes before production, using *Streptococcus* ssp. *thermophilus* and *Lactobacillus delbrueckii* ssp. *bulgaricus*.

It is worth noting that the higher levels of the actual number of viable cells encapsulated in the modified alginate matrix increased during storage of a fermented milk drink for 21 days, in comparison with the unprotected form of introduced probiotic cultures.

## 4. Conclusions

The effect of the composition and structure of a microcapsule made of biodegradable natural polymers on the preservation of the viability of immobilized bifidobacteria during oral delivery has been studied. In the sample of immobilized *Bifidobacterium bifidum* 791 in the structure of alginate hydrogel, the concentration of viable bifidobacteria decreased by 37.5% of the initial concentration when passing the model stomach environment (pH 1.2). The influence of resistant starch on the process of immobilization of bifidobacteria has been studied. Resistant starch in combination with alginate has a synergistic effect on gelation, providing additional protection for probiotic cells. To increase the stability of bifidobacteria, resistant starch was introduced into the composition of biodegradable microcapsules. The introduction of resistant starch into the structure of microcapsules improved the protection and preservation of the viability of microencapsulated bifidobacteria up to 87% of the initial concentration during the passage through model environments simulating the process of human digestion. The use of sodium carboxymethylcellulose 0.5% in the formation of a polymer microcapsule provided the maximum encapsulation efficiency of 93.2% and the maximum decay rate of microcapsules with bacteria of 95.7%. The morphological characteristics of microparticles were studied using scanning electron microscopy. The microparticles had an average diameter of 150 and 97 *μ*m. The concentration of bifidobacteria cells in a fermented milk drink containing encapsulated probiotics did not decrease below the recommended level (106–107 CFU/ml or g) during 21 days of storage.

## Data Availability

The data used to corroborate the results of this study are available from the respective author upon request.
